# Genomic Scanning of Inbreeding Depression for Litter Size in Two Varieties of Iberian Pigs

**DOI:** 10.3390/genes14101941

**Published:** 2023-10-15

**Authors:** Carlos Hervás-Rivero, Houssemeddine Srihi, David López-Carbonell, Joaquim Casellas, Noelia Ibáñez-Escriche, Sara Negro, Luis Varona

**Affiliations:** 1 Instituto Agroalimentario de Aragón (IA2), Universidad de Zaragoza, 50013 Zaragoza, Spain; chervas@unizar.es (C.H.-R.); davidlc@unizar.es (D.L.-C.); 2 Department Ciència Animal i dels Aliments, Universitat Autònoma de Barcelona, Bellaterra, 08193 Barcelona, Spain; 3 Instituto Universitario de Ciencia y Tecnología Animal, Universitat Politècnica de València, 46022 Valencia, Spain; 4 Programa de Mejora Genética “Castua”, INGA FOOD S. A. (Nutreco), 06200 Almendralejo, Spain

**Keywords:** Iberian pig, litter size, inbreeding depression

## Abstract

Inbreeding depression is expected to be more pronounced in fitness-related traits, such as pig litter size. Recent studies have suggested that the genetic determinism of inbreeding depression may be heterogeneous across the genome. Therefore, the objective of this study was to conduct a genomic scan of the whole pig autosomal genome to detect the genomic regions that control inbreeding depression for litter size in two varieties of Iberian pigs (Entrepelado and Retinto). The datasets consisted of 2069 (338 sows) and 2028 (327 sows) records of litter size (Total Number Born and Number Born Alive) for the Entrepelado and Retinto varieties. All sows were genotyped using the Geneseek GGP PorcineHD 70 K chip. We employed the Unfavorable Haplotype Finder software to extract runs of homozygosity (ROHs) and conducted a mixed-model analysis to identify highly significant differences between homozygous and heterozygous sows for each specific ROH. A total of eight genomic regions located on SSC2, SSC5, SSC7, SSC8, and SSC13 were significantly associated with inbreeding depression, housing some relevant genes such as FSHR, LHCGR, CORIN, AQP6, and CEP120.

## 1. Introduction

The most significant consequence of inbreeding in the phenotypic performance of livestock populations is the occurrence of inbreeding depression [1]. Theoretically, inbreeding depression arises from two genetic mechanisms, the impact from recessive mutations and the loss of contributions from of over-dominance genes [2]. This phenomenon is particularly evident in traits related to fitness, such as pig litter size [3,4]. Traditionally, inbreeding has been quantified using genealogical information [5]. However, the advent of high-throughput genotyping technologies has introduced a valuable tool for unraveling the genetic basis of inbreeding depression. Several studies [6,7,8] have indicated that its genetic determination is distributed unevenly across the genome.

One method widely used to detect identical-by-descent (IDB) genomic segments is to conduct runs of homozygosity (ROH) [9]. ROH are completely homozygous segments of an individual’s genome. Howard et al. [10] proposed a strategy for identifying genomic regions associated with inbreeding depression by contrasting the phenotypic performance of individuals carrying specific ROH with those lacking them, employing a mixed-model analysis [11].

In the context of Iberian pig populations, non-uniform effects of inbreeding depression across the genome were observed in a closed experimental flock of the Guadyerbas variety [7]. However, due to the great genetic diversity among the strains of the Iberian pig [12,13], variations in the genetic determinants of inbreeding depression may exist. Hence, the objective of this study is to investigate the genomic architecture of inbreeding depression effects in two commercial varieties of Iberian pigs (Entrepelado and Retinto). We also aim to pinpoint potential candidate genes located within the most relevant genomic regions.

## 2. Materials and Methods

The dataset utilized comprises 2069 records (pertaining to 338 sows) for the Entrepleado variety and 2028 records (related to 327 sows) for the Retinto for both TNB (Total Number Born) and NBA (Number Born Alive). In conjunction with this, a pedigree that contains genetically interconnected individuals was incorporated, with a total of 581 individuals for Entrepelado and 541 individuals for Retinto. The mean phenotypic performance values for TNB and NBA are summarized in Table 1.

Each sow was genotyped using the Geneseek GGP PorcineHD 70 K (Illumina Inc., San Diego, CA, USA) chip. Subsequent to genotyping, the genotypic data underwent filtration using PLINK 1.9 [14]. Filters were applied to ensure individual and SNP call rates exceeding 95%, with inclusion restricted to autosomal chromosomes. This process resulted in a collective sum of 57,450 SNP markers. Instances of missing genotypic data were rectified utilizing the FImpute 3. 0. software [15]. The allocation of SNP markers across the autosomal chromosomes in the Sscrofa 11.1 assembly is detailed in Table 2.

Firstly, we formulated a mixed linear model to assess the variance components and calculate the inbreeding depression through the gradient of a covariate associated with the percentage of individual heterozygosity, measured as the number of heterozygous SNPs per individual × 100 divided by the total number of SNPs.

The model we postulated for both varieties is as follows:

(1) y=fd+Xb+Th+Zu+Wp+e

where ***y*** represents the vector comprising phenotypic records (specifically TNB and NBA), ***f*** is a vector encompassing individual heterozygosity, and ***b*** is the vector of systematic effects, which incorporates order of parity at 5 levels (1st, 2nd, 3rd, 4th and beyond). Additionally, ***h*** is a vector of random herd–year–season, with 96 levels for Entrepelado and 113 for Retinto, ***u*** denotes the vector of additive genetic random effects, ***p*** is the permanent environmental sow effect and ***e*** stands for the vector of residuals. Moreover, *d* serves as a covariate of the relationship between individual heterozygosity and phenotypic performance. The matrices ***X***, ***T***, ***Z*** and ***W*** are the corresponding incidence matrices. The genomic relationships (***G***) among the additive genetic effects (***u***) were calculated using the single-step approach [16,17]. For the estimation of variance components, the average information residual maximum likelihood [18] was adopted, utilizing the blupf90+ software (http://nce.ads.uga.edu/wiki/doku.php?id=application_programs, accessed on 8 October 2023) [19].

Secondly, the Unfavorable Haplotype Finder software (https://github.com/jeremyhoward/Unfavorable-Haplotype-Finder, accessed on 8 October 2023) [10] was employed with the aim of selecting ROH. In this study, we defined ROH as a continuous sequence of homozygous genotypes spanning over 15 SNP markers. Additionally, we introduced a secondary criterion requiring that these ROHs be present in a minimum of 5% and a maximum of 95% of individuals within the population. However, we did not impose any constraints on the phenotypic traits of individuals, whether they carried the ROHs or not, as our primary objective was to identify all ROHs present in the populations. The algorithm’s details are expounded in [10].

Concluding this step, the blupf90+ software [19] was utilized to quantify the phenotypic impact associated with the presence or absence of each identified ROH. We solved a mixed model for each identified ROH. These models incorporated the previously estimated variance components and included systematic, permanent environmental, and additive genetic effects, along with an additional systematic effect related to the presence or absence of the ROH. The significance for this systematic effect was assessed using a one-sided *t*-test.

## 3. Results and Discussion

### 3.1. Variance Component Estimation

The results of the variance component estimation are presented in Table 3.

The estimates of the (co)variance components were similar to the ones provided by Srihi et al. [20], and they imply heritability estimates within the lower range of other estimates for white [21,22,23] and Iberian [24,25,26] pigs. It must be noted, however, that their impact on the ROH effect estimates are expected to be very low.

Given the estimates of the variance components, the estimates of the covariate with the percentage of heterozygosity were 0.055 ± 0.026 (*p* = 0.017) and 0.057 ± 0.028 (*p* = 0.021) for NBA and TNB in the case of Entrepelado and 0.077 ± 0.051 (*p* = 0.065) and 0.067 ± 0.050 (*p* = 0.090) for NBA and TNB in the case of Retinto. In all traits and populations, there was an increase in the litter size as the percentage of heterozygosity increased, leading to significant results for the Entrepelado population.

### 3.2. Runs of Homozygosity (ROH) Identification

**Figure 1:**
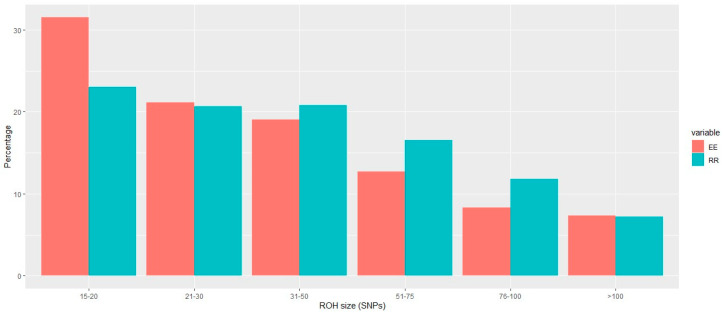
Distribution of the ROH sizes (according to SNP number) in the Entrepelado (EE) and Retinto (RR) populations.

**Figure 2:**
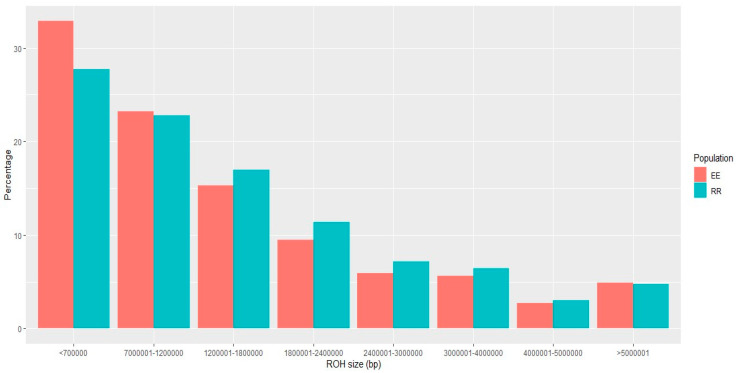
Distribution of the ROH sizes (according to base pairs) in the Entrepelado (EE) and Retinto (RR) populations.

We identified 43,188 and 35,175 runs of homozygosity (ROHs) consisting of more than 15 SNPs within the Entrepelado and the Retinto varieties, respectively. The ROH with the minimum length had 145,783 base pairs, and the larger ROH comprised 15,162,018 base pairs. Figure 1 and Figure 2 illustrate the distribution of ROH sizes according to the SNP number and base pairs, respectively.

These distributions highlight the prevalence of short ROHs in the Entrepelado population, in contrast to the right-skewed distribution observed in the Retinto population. This observation may suggest more recent inbreeding within the Retinto population. The average size of the ROHs in each population was 25.79 SNPs (±18.00) for Entrepelado and 35.96 SNPs (±24.30) for Retinto. Furthermore, the average percentage of an individual’s genome covered by ROHs, considering overlapping regions between ROHs, was 26.87% (±3.78%) for Entrepelado and 40.74% (±3.20%) for Retinto.

### 3.3. ROH Segments and Inbreeding Depression

**Figure 3:**
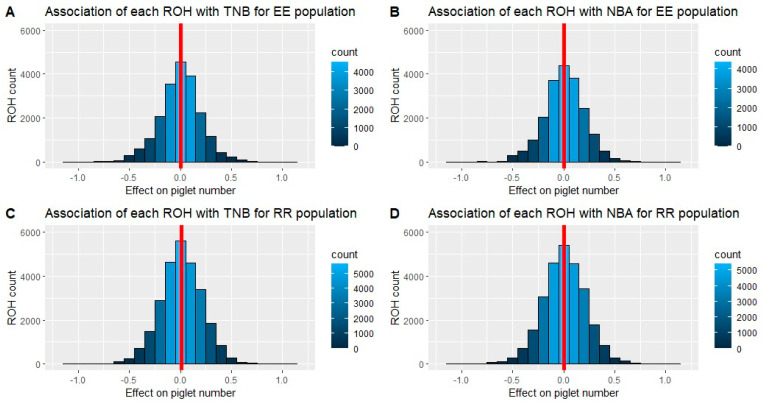
Distribution of the estimates of effects associated with the presence or absence of ROH for TNB (Total Number Born) and NBA (Number Born Alive) in Entrepelado and Retinto. Red lines represent the average effect of the ROH on TNB and NBA.

Among all the detected ROHs, we were able to identify 20,143 (Entrepelado) and 26,771 (Retinto) ROHs shared by at least 5% and at most 95% of individuals, composed of more than 15 SNPs, in which we expected to find that most of the variance was due to the ROH effect. Therefore, we solved 20,143 and 26,771 mixed-model equations for Entrepelado and Retinto, respectively. The objective was to obtain estimates of the effects related to the presence or absence of each specific ROH. The distributions of these effect estimates, pertaining to TNB and NBA, are illustrated in Figure 3 for both populations.

The average estimate of the effects was consistently close to zero across all scenarios, indicating that most of the ROHs were not associated with inbreeding depression. The genomic regions associated with inbreeding depression (*p* < 0.05) encompassed 1123 and 1533 runs of homozygosity (ROH) for NBA in Entrepelado and Retinto, respectively, while for TNB, they numbered 1197 and 1453 regions. These findings represent a proportion of significant ROHs that ranged from 5.4% (for RR and TNB) to 5.9% (for EE and TNB), slightly higher than what would be expected at random.

**Figure 4:**
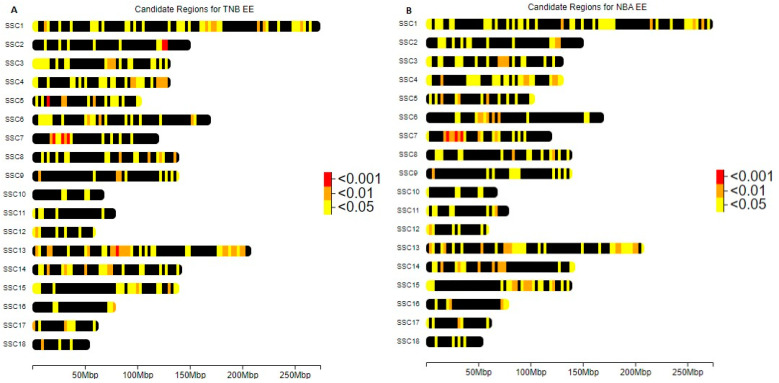
Distribution of ROHs with p-values lower than 0.05, 0.01 and 0.001 in Entrepelado (EE) population for (**A**) Total Number Born (TNB) and (**B**) Number Born Alive (NBA).

**Figure 5:**
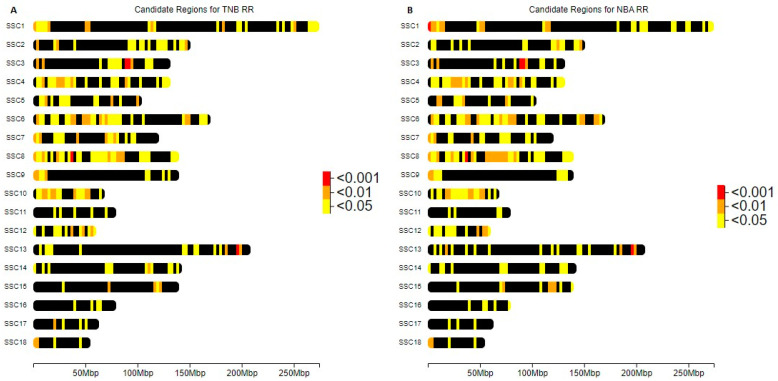
Distribution of ROHs with p-values lower than 0.05, 0.01 and 0.001 in Retinto (RR) population for (**A**) Total Number Born (TNB) and (**B**) Number Born Alive (NBA).

These significant ROHs exhibit a heterogeneous distribution across all chromosomes for both populations, as depicted in Figure 4 and Figure 5. Among these ROHs, 14 and 3 ROHs boast a particularly striking significance, with p-values below 0.001. The ROHs associated with *p*-values lower than 0.001 are presented in Table 4 and Table 5 for the Entrepelado and Retinto populations, respectively.

The regions identified in the NBA are also observed in the TNB results and are located on SSC7, having the lowest p-values. These regions span between 20,074,761 and 20,586,603 base pairs (bp), wherein proximity to QTLs associated with pig litter size [27,28] is noted. Within this region lies the GMNN (*Geminin DNA Replication Inhibitor*) gene, whose encoded protein plays an essential role in embryo development and implantation [29]. Additionally, in the genomic region spanning from 28,252,780 to 28,721,664 bp, we find the genes BAG2 (*BAG Cochaperone 2*) and RAB23 (*RAB23, Member RAS Oncogene Family*). The former is potentially linked to infertility, as the mediated inhibition of CHIP expression contributes to endometriosis [30], and the latter has been associated with litter size, as evidenced by GWAS in Bama Xiang pigs [31], and with failure during reproduction in puberty in a F2 population crossbreed of Duroc and Erhualian pigs [32]. Lastly, no associations with litter size were detected in the genomic region spanning from 80,682,896 to 81,329,857 bp.

The remaining regions with p-values lower than 0.001 in the TNB are distributed across SSC2 (7 ROHs), SSC5 (2 ROHs), and SSC13 (2 ROHs) in a contiguous manner. Within SSC2, spanning from 126,506,354 to 127,890,446 bp, we identified the CEP120 (*Centrosomal Protein 120*) gene, which has been associated with maternally derived aneuploidy [33]. In the SSC5 region (15,871,592–16,914,874 bp), we find the ATF1 (*Activating Transcription Factor 1*) gene, known to be involved in the estrogenic signaling pathway [34]. This region also includes AQP5 and AQP6 (*Aquaporin 5* and *Aquaporin 6*), which have been suggested as markers for male infertility in livestock [35]. AQP5 is overexpressed in granulosa cells and flattened follicle cells of the primordial follicles in the ovary and in the oviduct [36], while it is downregulated in pigs infected with Porcine Reproductive and Respiratory Syndrome [37]. We also identified RACGAP1 (*Rac GTPase-Activating Protein 1*), the inhibition of which is required in vitro for human embryonic trophoblast invasion into endometrial stromal cells [38]. Lastly, in the SSC13 region spanning from 80,594,858 to 81,329,857 bp, the only related gene found was CLSTN2 (*Calsyntenin 2*), which has been proposed as a potential candidate gene in Erhualian pigs [28,39] and in sheep after conducting a GWAS [40].

In the case of the RR population, we identified 17 ROHs with p-values lower than 0.001 in the NBA, 10 of which were shared with the TNB, as detailed in Table 4. The region with the lowest p-value is situated in SSC8, spanning from 37,024,885 to 37,966,306 base pairs (bp), with p-values of 6.77 × 10^−5^ in the case of NBA and 3.58 × 10^−4^ in the case of TNB. Additionally, within SSC8, there is another ROH ranging from 37,513,284 to 38,036,453 bp with a low p-value exclusive to NBA. Within this SSC8 region, several noteworthy genes are located, including GABRB1 (*γ-Aminobutyric Acid Type A Receptor Subunit Beta1*), which plays a role in inhibiting GnRH neurons. This inhibition is essential for the production of the GnRH hormone, which, in turn, is crucial for the synthesis of LH (*luteinizing hormone*) and FSH (*follicle-stimulating hormone*), both of which are crucial for reproduction [41,42,43]. CORIN (*Corin, Serine Peptidase*) is up-regulated in the decidua of the pregnant uterus, which suggests a potential role during pregnancy [44], and it has been proposed as a candidate gene for calving easiness in dairy and beef cattle [45]. This SSC8 region is also associated with QTLs linked to reproduction traits, such as litter size in the Chinese Erhualian pig breed [28] and the number of stillborn piglets in Shaziling pigs [46]. Furthermore, regions on SSC3 and SSC13 were shared by the NBA and TNB and contained genes like FSHR (*follicle-stimulating hormone receptor*) and LHCGR (*luteinizing hormone receptor*), both critical in regulating female reproductive processes. Additionally, GTF2A1L (*General Transcription Factor IIA Subunit 1 Like*) may play an important role in testis biology and male infertility [47]. On SSC13, in positions 196, 187, 718–196, 471 and 450, near a QTL for litter size [28], lies the USP16 (*Ubiquitin-Specific Peptidase 16*) gene, responsible for regulating embryonic stem cell gene expression [48]. In this region, CFAP298 (*Cilia- and Flagella-Associated Protein 298*) has been described, with a mutation known to cause infertility in human patients [49]. Lastly, there is a region with p-values lower than 0.001 in the NBA at SSC1 spanning from 632,758 to 2,160,998 bp, although no specific relationships with reproductive traits were identified.

## 4. Conclusions

The results of this study indicate the presence of inbreeding depression in the litter size traits of two strains of Iberian pigs. Furthermore, the distribution of the inbreeding depression effects is heterogeneous along the genome, and the architecture of inbreeding depression differs between populations. Additionally, we were able to identify eight genomic regions significantly associated with inbreeding depression that contain several relevant genes, such as FSHR, LHCGR, CORIN, AQP6 and CEP120.

## Figures and Tables

**Table 1:** Mean and standard deviation (between brackets) of TNB (Total Number Born) and NBA (Number Born Alive) in the Entrepelado and Retinto varieties.

Population	TNB	NBA
Entrepelado	7.96 (1.93)	7.70 (1.88)
Retinto	8.27 (2.18)	7.99 (2.17)

**Table 2:** Chromosome (SSC), number of SNP markers (Nm) and base pairs covered (bp).

SSC	Nm	bp
1	5442	274,315,671
2	3713	151,610,480
3	3308	132,657,669
4	3557	130,773,976
5	2716	104,477,606
6	4368	170,802,600
7	3563	121,758,423
8	3324	138,930,735
9	3513	139,386,589
10	2477	69,319,537
11	2178	79,072,521
12	2295	60,834,034
13	4146	208,240,759
14	3812	141,719,266
15	3281	140,404,164
16	2205	79,282,526
17	1968	63,391,207
18	1607	55,752,892

**Table 3:** Restricted maximum likelihood estimates (and sampling variance) of the additive ($\sigma_{a}^{2}$), permanent environmental ($\sigma_{p}^{2}$), herd–year–season ($\sigma_{h}^{2}$) and residual ($\sigma_{e}^{2}$) variance.

	Entrepelado		Retinto	
	TNB	NBA	TNB	NBA
$\sigma_{a}^{2}$	0.145 (0.085)	0.098 (0.071)	0.165 (0.092)	0.225 (0.110)
$\sigma_{p}^{2}$	0.366 (0.097)	0.341 (0.089)	0.283 (0.11)	0.291 (0.116)
$\sigma_{h}^{2}$	0.170 (0.545)	0.129 (0.071)	0.317 (0.085)	0.172 (0.061)
$\sigma_{e}^{2}$	2.901 (0.101)	2.853 (0.099)	3.908 (0.138)	3.796 (0.134)

**Table 4:** Chromosome (SSC), base pair position (bp), and effect on number of piglets for Number Born Alive (piglet NBA), effect on number of piglets for Total Number Born (piglet TNB), *p*-value for Number Born Alive (*p*-value NBA), *p*-value for Total Number Born (*p*-value TNB) and candidate genes within the genomic regions for Entrepelado population.

SSC	bp(c)	Piglets (NBA)	Piglets (TNB)	*p*-Value (NBA)	*p*-Value (TNB)	Genes
SSC2	126,506,354–126,841,331	−0.5428	−0.6264	2.23 × 10^−3^	8.83 × 10^−4^	CEP120
	126,516,022–126,857,203	−0.5358	−0.6239	1.28 × 10^−3^	4.08 × 10^−4^	
	126,562,142–126,971,814	−0.4838	−0.5840	3.46 × 10^−3^	9.82 × 10^−4^	
	126,700,457–127,000,238	−0.4935	−0.5904	2.69 × 10^−3^	7.87 × 10^−4^	
	126,777,258–127,080,032	−0.4838	−0.5840	3.46 × 10^−3^	9.82 × 10^−4^	
	127,096,117–127,764,704	−0.6088	−0.7179	2.75 × 10^−3^	9.18 × 10^−4^	
	127,492,532–127,890,446	−0.6305	−0.7393	1.70 × 10^−3^	5.36 × 10^−4^	
SSC5	15,871,592–16,914,874	−0.6141	−0.7758	5.00 × 10^−3^	9.12 × 10^−4^	ATF1 AQP5 AQP6 RACGAP1
	16,134,195–16,852,941	−0.5377	−0.7575	1.10 × 10^−2^	9.73 × 10^−4^	
SSC7	20,074,761–20,586,603	−0.5995	−0.5869	7.23 × 10^−5^	2.03 × 10^−4^	GMNN
	28,252,780–28,721,664	−0.6691	−0.6795	3.54 × 10^−5^	6.34 × 10^−5^	
	35,594,623–36,062,243	−0.6175	−0.6308	6.97 × 10^−4^	9.86 × 10^−4^	BAG2 RAB23
	20,074,761–20,586,603	−0.5995	−0.5869	7.23 × 10^−5^	2.03 × 10^−4^	
SSC13	80,594,858–81,214,645	−0.4839	−0.5679	2.28 × 10^−3^	7.63 × 10^−4^	CLSTN2
	80,682,896–81,329,857	−0.4929	−0.5693	1.72 × 10^−3^	6.53 × 10^−4^	

**Table 5:** Chromosome (SSC), base pair position (bp), effect on number of piglets for Number Born Alive (piglet NBA), effect on number of piglets for Total Number Born (piglet TNB), *p*-value and FDR for Number Born Alive (*p*-value/FDR NBA), *p*-value and FDR for Total Number Born (*p*-value/FDR TNB), and candidate genes within the genomic regions for Retinto population.

SSC	bp(c)	Piglets (NBA)	Piglets (TNB)	*p*-Value (NBA)	*p*-Value (TNB)	Genes
SSC1	632,758–1,869,413	−0.9609	−0.7750	1.35 × 10^−4^	1.41 × 10^−3^	
	734,657–1,869,413	−0.9609	−0.6383	1.35 × 10^−4^	4.82 × 10^−3^	
	989,159–1,358,337	−0.9609	−0.7750	1.35 × 10^−4^	1.41 × 10^−3^	
	1,189,180–1,471,069	−0.8987	−0.7750	2.31 × 10^−4^	1.41 × 10^−3^	
	1,189,180–2,160,998	−0.8174	−0.7244	5.59 × 10^−4^	2.06 × 10^−3^	
SSC3	88,968,396–91,465,748	−0.5929	−0.5525	7.53 × 10^−4^	1.26 × 10^−3^	FSHR LHCGR GTF2A1L
	90,580,151–91,903,672	−0.6761	−0.6322	1.69 × 10^−4^	3.08 × 10^−4^	
	91,082,368–92,692,939	−0.6931	−0.6421	1.60 × 10^−4^	3.29 × 10^−4^	
	91,240,698–91,903,672	−0.6388	−0.6003	2.88 × 10^−4^	4.70 × 10^−4^	
	91,265,546–92,395,629	−0.6761	−0.6322	1.69 × 10^−4^	3.08 × 10^−4^	
	91,381,281–92,692,939	−0.6608	−0.6010	2.64 × 10^−4^	6.37 × 10^−4^	
	91,381,281–92,437,314	−0.6458	−0.5931	2.75 × 10^−4^	5.88 × 10^−4^	
	91,381,281–91,966,239	−0.6102	−0.5632	4.53 × 10^−4^	8.66 × 10^−4^	
SSC8	37,024,885–37,966,306	−1.0159	−0.8876	6.77 × 10^−5^	3.58 × 10^−4^	GABRB1 CORIN
	37,513,284–38,036,453	−0.8992	−0.7349	2.59 × 10^−4^	2.00 × 10^−3^	
SSC13	196,187,718−196,460,966	−0.6757	−0.6677	9.06 × 10^−4^	8.51 × 10^−4^	USP16 CFAP298
	196,216,549–196,471,450	−0.7089	−0.6826	3.89 × 10^−4^	4.98 × 10^−4^	

## Data Availability

The dataset used in this study will be available upon reasonable request from the corresponding author (lvarona@unizar.es).
